# EMOTIONAL RESPONSE TO THE COVID-19 PANDEMIC: NURSING HOME RESIDENTS’ PERSPECTIVES

**DOI:** 10.1093/geroni/igad104.2264

**Published:** 2023-12-21

**Authors:** Ashley Woods, Amy Vogelsmeier, Lori Popejoy, Steven Miller, Alisha Johnson, Roy Thompson, Amy Trueblood

**Affiliations:** University of Missouri, Columbia, Missouri, United States; University of Missouri, Columbia, Missouri, United States; University of Missouri, Columbia, Missouri, United States; University of Missouri, Columbia, Missouri, United States; University of Missouri, Columbia, Missouri, United States; University of Missouri, Columbia, Missouri, United States; University of Missouri, Columbia, Missouri, United States

## Abstract

Nursing home residents were at the forefront of challenges imposed by the COVID-19 pandemic, including being forced into extended periods of isolation, yet little is known about their emotional response. Therefore, the purpose of this study is to describe residents’ emotional responses to the COVID-19 pandemic, including their response to isolation. Twenty-eight residents residing in 22 Missouri nursing homes during the pandemic participated in semi-structured interviews as part of a larger mixed-methods study to understand nursing homes’ response to COVID-19. Interviews were transcribed and analyzed by a team of PhD researchers and doctoral students using a qualitative content analysis approach. Of the 28 resident interviews, most were more concerned about their families, other residents, and staff becoming infected rather than themselves. Many cited their lack of concern was due to trust in nursing home staff to keep them safe. Regarding isolation, a few described being angry about separation from family and friends. Nevertheless, most understood that the restrictions were about “doing what had to be done” and that isolation was “for the greater good.” The majority also responded positively about their relationships with staff during isolation and happiness at the return of visitors after isolation restrictions were lifted. Overall, residents’ emotional responses during the COVID-19 pandemic varied but primarily reflected their concern for others rather than themselves. Their concern for themselves may have been allayed because of their trust in nursing home staff. Their response to isolation seemed rooted in their understanding of the need to protect themselves and others.

